# Drug-induced cataract: a real-world study based on the food and drug administration adverse event reporting system database

**DOI:** 10.3389/fmed.2026.1758892

**Published:** 2026-01-16

**Authors:** Xianfen Cao, Xiaoping Zhou, Shinan Wu, Jing Zeng, Yulun Ou, Qing Zhou

**Affiliations:** 1 Department of Ophthalmology, The First People’s Hospital of Chenzhou, Chenzhou, Hunan, China; 2 Department of Ophthalmology, The First Affiliated Hospital of Jinan University, Guangzhou, China; 3 Eye Institute of Xiamen University, School of Medicine, Xiamen University, Xiamen, Fujian, China; 4 Ophthalmic Center, The Second Affiliated Hospital of Guangzhou Medical University, Guangzhou, China

**Keywords:** cataract, drug induction time, drug-induced risk, FAERS, pharmacovigilance

## Abstract

**Purpose:**

This study aims to investigate the risk of drug-induced cataract and examine its epidemiological patterns using real-world data.

**Methods:**

Data from the FDA Adverse Event Reporting System (FAERS), spanning January 2004 to December 2024, were analyzed. A disproportionality analysis was conducted on the FAERS database using four quantitative measures—reporting odds ratio (ROR), proportional reporting ratio (PRR), Bayesian confidence propagation neural network (BCPNN), and multi-item gamma Poisson shrinker (MGPS)—to identify potential safety signals. The study categorized the identified cataract-induced drugs by risk level and quantitatively compared the time to onset across these categories.

**Results:**

A total of 671 drugs were reported to be associated with cataract in the FAERS database. Disproportionality analysis identified 64 drugs with a significant risk of cataract formation. The primary therapeutic classes included hormonal, oncological, and ophthalmic medications, along with drugs acting on the nervous system. The highest-risk drugs identified were omidenepag isopropyl, clobazam, and nitisinone, with BCPNN scores of 7.69, 7.36, and 6.02, respectively. Ophthalmic medications showed the shortest mean onset time for drug-induced cataract, averaging 120.29 days. The majority of affected individuals were female (67.59%) and elderly (mean age 63.85 ± 14.54 years).

**Conclusion:**

This study provides real-world evidence regarding the risk of drug-induced cataract, offering empirical support for preventive strategies and informed clinical decision-making.

## Introduction

Cataract development is characterized by the clouding of the eye’s crystalline lens, resulting from the aggregation and precipitation of proteins, ultimately leading to a progressive decline in visual quality (1). As the leading cause of blindness globally, cataract disproportionately impact populations in middle- and low-income countries (2). Current estimates indicate that over 10 million people are blind due to cataract, with an additional 35 million experiencing moderate to severe vision impairment, making it a significant global health challenge (3).

Cataracts are classified by their etiology into several subtypes, including age-related, congenital, secondary, traumatic, radiation-induced, and drug-induced (4, 5). Among these, drug-induced cataract represent a clinically significant, yet often underrecognized, contributor to visual impairment. These cataract develop as a result of prolonged exposure to specific pharmacological agents, with over 70 medications identified as potential risk factors (6, 7). A critical clinical consideration is that, in early or mild cases, drug-induced cataract may be reversible, or their progression can be halted if the causative medication is identified and discontinued promptly. Therefore, the early identification of drugs that can induce cataract is crucial in clinical practice. Historically, prior to comprehensive databases like the FDA Adverse Event Reporting System (FAERS), knowledge of drug-induced cataract was based largely on isolated case reports and limited surveillance (8). Although these methods established common associations, they had notable limitations, including the systematic underreporting of rare medications, substantial publication delays, and the potential for false-positive signals in studies with limited statistical power.

This study aims to systematically evaluate drug-induced cataract using a large-scale real-world dataset derived from the FAERS database, with the additional goal of identifying risks associated with pharmacological agents not yet recognized as causing cataract. Our objectives are to identify drugs associated with cataract in clinical practice, quantify their specific risks, and determine the typical onset time following drug initiation.

## Methods

### Data source

**Figure 1:**
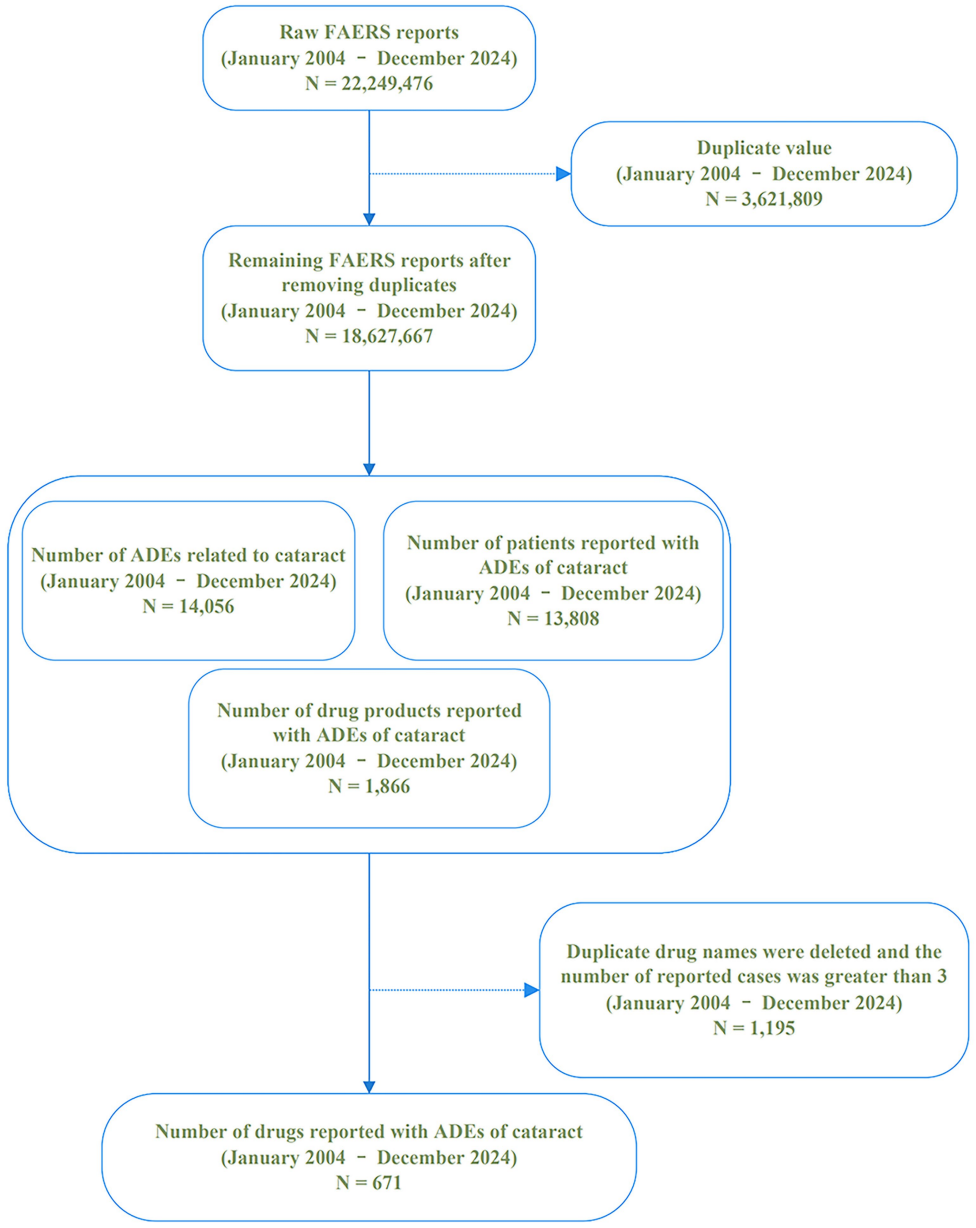
Flowchart of the data cleaning pipeline for drug-induced cataract data from the FAERS database.

This study utilized data extracted from the FAERS, covering the period from January 1, 2004, to December 31, 2024. These datasets are publicly available for download on the FDA’s official website. The database consolidates voluntary adverse drug reaction (ADR) reports submitted by global stakeholders, including healthcare professionals, pharmaceutical companies, and consumers. To strengthen the validity of the analysis, only reports submitted by physicians and pharmacists were included. In accordance with FDA deduplication standards, reports were organized by PRIMARYID, CASEID, and FDA_DT. For instances with duplicate combinations of CASEID and FDA_DT, only the record with the highest PRIMARYID and the most recent FDA_DT was retained, ensuring the most up-to-date entry for each case (9). Between January 2004 and December 2024, the database accumulated 22,249,476 case entries in its unprocessed form. After deduplication using primary ID identifiers, 18,627,667 entries remained for analysis. Within this dataset, 14,056 cataract-associated adverse event reports were identified, corresponding to 13,808 unique subjects experiencing drug-induced cataract reactions linked to 1,866 pharmaceutical agents. After applying exclusion criteria (removing drugs with fewer than three reported cases and consolidating duplicate brand names), 671 distinct medications were retained for final analysis. The data cleaning process is systematically presented in Figure 1.

### Identification of ADRs

This study utilized the Medical Dictionary for Regulatory Activities (MedDRA) to define ADRs (MedDRA® version 20.0) (10). Adverse events were encoded using MedDRA Preferred Terms (PTs), and related PTs were identified through standardized MedDRA queries (SMQs) specific to cataract. In this study, only PTs of narrow scope were employed (11).

### Statistical analysis

Signal detection was conducted using disproportionality analysis, which incorporated four complementary statistical methods: the reporting odds ratio (ROR), proportional reporting ratio (PRR), Bayesian confidence propagation neural network (BCPNN), and multi-item gamma Poisson shrinker (MGPS). ROR and PRR were selected for their high sensitivity and computational simplicity; however, these measures can be unstable in sparse data and may generate false-positive signals due to random variability. To mitigate this limitation, Bayesian shrinkage methods (BCPNN and MGPS) were applied, as they provide higher specificity in the context of rare events (12). This study therefore centered on adverse event meeting all four algorithm criteria. All methods were implemented within a standard contingency table framework (Tables 1, 2). The criteria for positive signals were as follows: (1) For ROR, a value ≥3 with the lower limit of the 95% confidence interval (CI) > 1; (2) For PRR, a value ≥3 with the lower limit of the 95% CI > 1; (3) For BCPNN, the lower limit of the information component (IC_025_) > 0; (4) For MGPS, the standard is EBGM_05_ > 2 and a > 0. For the BCPNN method, signal strength was classified based on IC_025_ values: signals were considered weak (IC_025_ 0–1.5), medium (IC_025_ 1.5–3), or strong (IC_025_ > 3). Additionally, Quartiles of drug-induced event onset times were calculated to analyze temporal patterns across drugs. A multivariable logistic regression was conducted on the top 20 signal drugs to assess risk profiles, adjusting for confounders like age, gender, reporting country, administration route, and drug indications. All analyses were conducted using SPSS (v26.0), GraphPad Prism (v10.1.2), Excel 2019, and R (v4.2.2). For visualization and statistical workflows in R, the following packages were used: ggplot2, ggrepel, dplyr, and DescTools. A two-sided *p* < 0.05 was considered statistically significant.

**Table 1:** Four-grid table of disproportionality analysis method.

Item	Target adverse events	All other adverse events	Total
Target drugs	a	b	a + b
All other drugs	c	d	c + d
Total	a + c	b + d	a + b + c + d

A contingency table for the calculation formula of the proportion imbalance analysis.

**Table 2:** Principle of disproportionality analysis and standard of signal detection.

Methods	Calculation formula	Inclusion standard of positive signal
ROR	$\mathrm{ROR}=\frac{\;(a/c)}{\;(b/d)}$	a ≥ 3 and 95%CI > 1
	$\mathrm{SE}\;(\text{lnROR})=\sqrt{\;(\frac{1}{a}+\frac{1}{b}+\frac{1}{c}+\frac{1}{d})}$	
	$95\%\mathrm{CI}=e^{\ln\;(\mathrm{ROR})\pm 1.96\sqrt{\frac{1}{a}+\frac{1}{b}+\frac{1}{c}+\frac{1}{d}}}$	
PRR	$\mathrm{PRR}=\frac{a/(a+b)}{c/(c+d)}$	a ≥ 3 and 95%CI > 1
	$\mathrm{SE}\;(\text{lnPRR})=\sqrt{\;(\frac{1}{a}-\frac{1}{a+b}+\frac{1}{c}-\frac{1}{c+d})}$	
	$95\%\mathrm{CI}=e^{\ln\;(\mathrm{PRR})\pm 1.96\sqrt{\;(\frac{1}{a}-\frac{1}{a+b}+\frac{1}{c}-\frac{1}{c+d})}}$	
BCPNN	$\mathrm{IC}=\log_{2}\frac{a\;(a+b+c+d)}{\;(a+b)\;(a+c)}$	No signal (−): IC_025_ ≤ 0 Low signal (+): 0 < IC_025_ ≤ 1.5 Medium signal (++): 1.5 < IC_025_ ≤ 3 High signal (+++): IC_025_ > 3.
	${\mathrm{IC}}_{025}=\log_{2}\frac{\;(a+\gamma 11)\;(a+b+c+d+\alpha )\;(a+b+c+d+\beta )}{\;(a+b+c+d+\gamma )\;(a+b+\alpha 1)\;(a+c+\beta 1)}$	
	$\begin{array}{l} V\;(\mathrm{IC})=\frac{1}{\;{\left(\ln2\right)}^{2}}\{[\frac{\;(a+b+c+d)-a+\gamma -\gamma 11}{\;(a+\gamma 11)\;(1+a+b+c+d+\gamma )}]\\ +[\frac{\;(a+b+c+d)-(a+b)+a-\alpha 1}{\;(a+b+\alpha 1)\;(1+a+b+c+d+\alpha )}]\\ +[\frac{\;(a+b+c+d)-(a+c)+\beta -\beta 1}{\;(a+c+\beta 1)\;(1+a+b+c+d+\beta )}] \end{array}$	
	$\gamma =\gamma 11\frac{\;(a+b+c+d+\alpha )\;(a+b+c+d+\beta )}{\;(a+b+\alpha 1)\;(a+c+\beta 1)}$	
	$\mathrm{IC}-2\mathrm{SD}=E\;(\mathrm{IC})-2\sqrt{V\;(\mathrm{IC})}$	
	Where α1 = β1 = 1; α = β = 2; γ11=1	
MGPS	$\text{EBGM}=\frac{a\;(a+b+c+d)}{\;(a+c)\;(a+b)}$	EBGM05 > 2 and a > 0
	$\text{EBGM}05=e^{\ln\;(\text{EBGM})-\sqrt[{2}]{1.64\;(\frac{1}{a}+\frac{1}{b}+\frac{1}{c}+\frac{1}{d})}}$	

ROR, reporting odds ratio; PRR, proportional reported ratio; BCPNN, Bayesian confidence propagation neural network; MGPS, multi-item gamma Poisson shrinker; CI, confidence interval; IC, information component.

## Results

### Baseline characteristics of subjects

**Figure 2:**
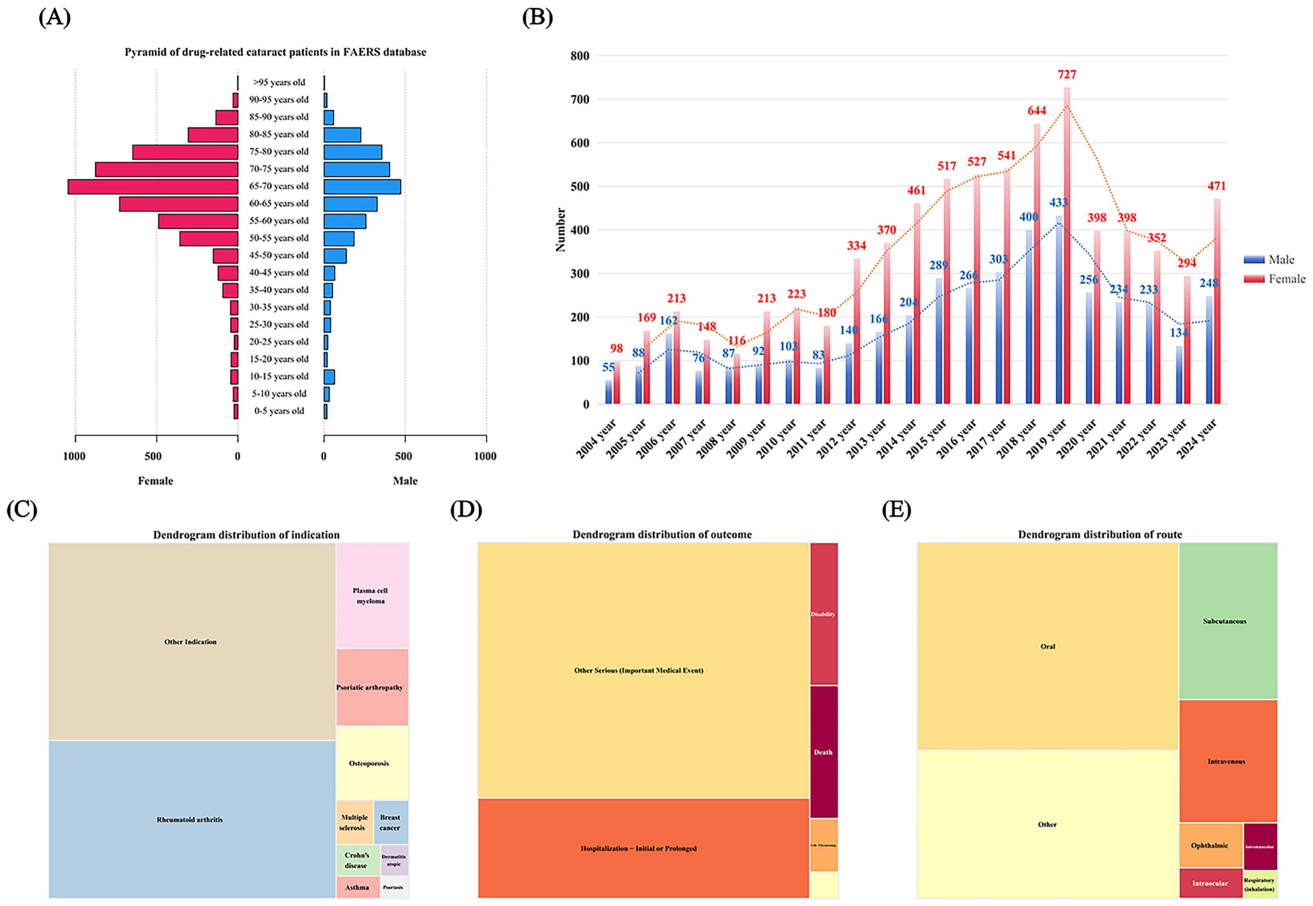
Distribution of baseline data for patients reporting adverse events of cataract in the FAERS database. **(A)** Patient age distribution by gender. **(B)** Reporting trend of adverse events. **(C)** Distribution of indication. **(D)** Distribution of patient outcomes. **(E)** Profile of drug administration routes.

A total of 13,808 subjects with reported ADRs associated with cataract were included in this study. The mean age of participants was 63.85 ± 14.54 years, with a predominance of females (67.59%) (Figure 2A). Since 2004, the reported incidence of drug-induced cataract has shown a rising epidemiological trend, peaking in 2019, with a higher prevalence observed in females compared to males (Figure 2B). The primary therapeutic indications were “other indications” (67.23%, *n* = 67,230), rheumatoid arthritis (17.99%, *n* = 1,632), and plasma cell myeloma (9.09%, *n* = 276) (Figure 2C). Among reports of drug-induced cataract, the most common serious clinical outcomes were hospitalization (38.18%, *n* = 1,159) and other serious conditions (48.29%, *n* = 1,466). Notably, fatal (4.48%, *n* = 270) and disabling (5.11%, *n* = 155) outcomes were also reported. It is critical to note that these outcomes may be attributed to co-reported adverse events rather than cataract themselves (Figure 2D). The United States, Canada, and Japan accounted for the highest number of reported cases (Figure 2E). Additional details are provided in Figure 2 and Table 3.

**Table 3:** Baseline characteristics of patients with drug-induced cataract.

Variable	Total
Age	63.85 ± 14.54
Weight	74.72 ± 20.89
Gender	
Female	2,052 (67.59)
Male	984 (32.41)
Reporter	
Physician	2,078 (68.45)
Other health-professional	763 (25.13)
Pharmacist	195 (6.42)
Country	
United States	1,625 (53.52)
Other Country	455 (14.99)
Canada	269 (8.86)
Japan	214 (7.05)
Germany	129 (4.25)
France	123 (4.05)
Brazil	99 (3.26)
United kingdom	83 (2.73)
Australia	19 (0.63)
Spain	11 (0.36)
Italy	9 (0.30)
Route	
Oral	1,679 (55.30)
Other	530 (17.46)
Subcutaneous	366 (12.06)
Intravenous	314 (10.34)
Ophthalmic	68 (2.24)
Intramuscular	43 (1.42)
Intraocular	18 (0.59)
Respiratory (inhalation)	18 (0.59)
Outcome	
Other Serious (Important Medical Event)	1,466 (48.29)
Hospitalization – Initial or Prolonged	1,159 (38.18)
Disability	155 (5.11)
Death	136 (4.48)
Life-threatening	80 (2.64)
Required Intervention to Prevent Permanent Impairment/Damage	39 (1.28)
Congenital Anomaly	1 (0.03)
Indication	
Other Indication	2,041 (67.23)
Rheumatoid arthritis	1,632 (17.99)
Plasma cell myeloma	276 (9.09)
Osteoporosis	194 (6.39)
Multiple sclerosis	60 (1.98)
Breast cancer	56 (1.84)
Crohn’s disease	50 (1.65)
Psoriatic arthropathy	175 (1.27)
Asthma	36 (1.19)
Dermatitis atopic	32 (1.05)
Psoriatic arthropathy	28 (0.92)
Psoriasis	23 (0.76)

### Distribution of drug categories associated with drug-induced cataract

In the disproportionality analysis of 671 drugs linked to cataract-related adverse reactions, positive signals were detected for 64 medications. The DrugBank database was used to retrieve brand/generic drug names and their respective mechanisms of action. Among the 64 signal-positive drugs, the distribution by therapeutic class was as follows: hormonal medications (16, 25%), oncological drugs (15, 23.4%), ophthalmic medications (6, 9.4%), nervous system agents (6, 9.4%), and other drug classes (21, 32.8%). The therapeutic classes and specific actions of these drugs are detailed in Table 4.

**Table 4:** Disproportionality analysis of positive signal drugs associated with cataract.

Drug name	Classification	ROR (95%CI)	PRR (95%CI)	MGPS (95%CI)	BCPNN (95%CI)	PRR (*X*^2^)	*p* value
Budesonide	Hormonal medication	15.15 (9.11–25.2)	15 (14.5–15.51)	14.99 (9.79–22.95)	3.91 (2.24–5.57)	15 (195.99)	<0.001
Conjugated estrogens	Hormonal medication	4.51 (2.94–6.92)	4.5 (4.07–4.92)	4.49 (3.14–6.43)	2.17 (0.5–3.83)	4.5 (57.04)	<0.001
Corticotropin	Hormonal medication	4.55 (2.17–9.57)	4.54 (3.8–5.28)	4.54 (2.44–8.45)	2.18 (0.52–3.85)	4.54 (19.35)	<0.001
Dexamethasone	Hormonal medication	29.09 (22.37–37.83)	28.55 (28.3–28.81)	28.44 (22.83–35.43)	4.83 (3.16–6.5)	28.55 (1510.43)	<0.001
Fluocinolone acetonide	Hormonal medication	17.14 (9.92–29.63)	16.96 (16.42–17.5)	16.94 (10.72–26.78)	4.08 (2.41–5.75)	16.96 (195.19)	<0.001
Fluticasone furoate	Hormonal medication	13.21 (6.28–27.81)	13.1 (12.37–13.84)	13.1 (7.03–24.42)	3.71 (2.04–5.38)	13.1 (78.28)	<0.001
Fluticasone propionate	Hormonal medication	5.66 (3.47–9.26)	5.65 (5.16–6.14)	5.64 (3.74–8.51)	2.5 (0.83–4.16)	5.65 (61.16)	<0.001
Methylprednisolone	Hormonal medication	5.46 (3.09–9.62)	5.44 (4.87–6)	5.44 (3.38–8.74)	2.44 (0.78–4.11)	5.44 (43.47)	<0.001
Mometasone furoate	Hormonal medication	6.47 (4.12–10.15)	6.44 (6–6.89)	6.44 (4.41–9.39)	2.69 (1.02–4.35)	6.44 (87.34)	<0.001
Pasireotide	Hormonal medication	4.64 (1.74–12.37)	4.62 (3.65–5.6)	4.62 (2.03–10.51)	2.21 (0.54–3.88)	4.62 (11.37)	0.005
Prednisolone	Hormonal medication	4.21 (1.89–9.38)	4.2 (3.4–5)	4.2 (2.15–8.21)	2.07 (0.4–3.74)	4.2 (14.64)	0.001
Prednisolone acetate	Hormonal medication	13.76 (8.53–22.19)	13.64 (13.17–14.11)	13.63 (9.13–20.32)	3.77 (2.1–5.44)	13.64 (199.03)	<0.001
Prednisolone phosphate	Hormonal medication	36.78 (17.37–77.87)	35.91 (35.18–36.65)	35.9 (19.16–67.24)	5.17 (3.49–6.84)	35.91 (237.63)	<0.001
Triamcinolone	Hormonal medication	6.65 (5.23–8.46)	6.63 (6.39–6.87)	6.6 (5.4–8.07)	2.72 (1.06–4.39)	6.63 (318.72)	<0.001
Clobazam	Nervous system medication	185.16 (55.75–614.98)	164.7 (163.63–165.77)	164.66 (60.31–449.57)	7.36 (5.61–9.12)	164.7 (488.34)	<0.001
Fluvoxamine	Nervous system medication	5.22 (1.68–16.21)	5.2 (4.07–6.33)	5.2 (2.01–13.43)	2.38 (0.71–4.05)	5.2 (10.18)	0.011
Imipramine	Nervous system medication	19.81 (11.2–35.02)	19.56 (19–20.12)	19.54 (12.13–31.48)	4.29 (2.62–5.96)	19.56 (211.28)	<0.001
Quetiapine	Nervous system medication	4.07 (3.29–5.02)	4.06 (3.85–4.27)	4.04 (3.39–4.82)	2.01 (0.35–3.68)	4.06 (199.41)	<0.001
Sumatriptan	Nervous system medication	7.27 (2.34–22.62)	7.24 (6.11–8.37)	7.24 (2.8–18.71)	2.86 (1.19–4.53)	7.24 (16.15)	0.001
Vutrisiran	Nervous system medication	19.37 (8.65–43.34)	19.13 (18.33–19.93)	19.12 (9.75–37.52)	4.26 (2.59–5.93)	19.13 (103.12)	<0.001
Anastrozole	Oncological medication	4.02 (2.53–6.38)	4.01 (3.55–4.47)	4 (2.72–5.9)	2 (0.34–3.67)	4.01 (40.61)	<0.001
Belantamab mafodotin	Oncological medication	11.38 (8.96–14.45)	11.3 (11.06–11.53)	11.25 (9.21–13.74)	3.49 (1.83–5.16)	11.3 (635.56)	<0.001
Bexarotene	Oncological medication	10.68 (4.79–23.85)	10.61 (9.82–11.41)	10.61 (5.42–20.78)	3.41 (1.74–5.08)	10.61 (52.27)	<0.001
Dutasteride	Oncological medication	4.61 (2.2–9.69)	4.6 (3.86–5.34)	4.6 (2.47–8.56)	2.2 (0.53–3.87)	4.6 (19.73)	<0.001
Enfortumab vedotin	Oncological medication	5.03 (2.85–8.87)	5.02 (4.45–5.58)	5.01 (3.12–8.06)	2.33 (0.66–3.99)	5.02 (38.59)	<0.001
Erdafitinib	Oncological medication	18.32 (10.6–31.66)	18.11 (17.57–18.65)	18.09 (11.44–28.59)	4.18 (2.51–5.85)	18.11 (210.04)	<0.001
Goserelin	Oncological medication	4.98 (1.87–13.29)	4.97 (3.99–5.94)	4.96 (2.18–11.29)	2.31 (0.64–3.98)	4.97 (12.67)	0.003
Lenalidomide	Oncological medication	4.71 (4.51–4.93)	4.7 (4.66–4.75)	4.08 (3.93–4.24)	2.03 (0.36–3.7)	4.7 (5715.59)	<0.001
Letrozole	Oncological medication	3.53 (2.3–5.41)	3.52 (3.09–3.95)	3.52 (2.46–5.03)	1.81 (0.15–3.48)	3.52 (37.84)	<0.001
Mirvetuximab soravtansine	Oncological medication	44.14 (24.85–78.39)	42.89 (42.33–43.45)	42.85 (26.5–69.3)	5.42 (3.75–7.09)	42.89 (490.85)	<0.001
Pomalidomide	Oncological medication	3.7 (3.37–4.07)	3.7 (3.6–3.79)	3.61 (3.34–3.9)	1.85 (0.19–3.52)	3.7 (866.39)	<0.001
Raloxifene	Oncological medication	5.37 (3.38–8.54)	5.36 (4.9–5.82)	5.35 (3.63–7.88)	2.42 (0.75–4.09)	5.36 (63.74)	<0.001
Rituximab	Oncological medication	6.6 (4.87–8.95)	6.58 (6.28–6.88)	6.56 (5.09–8.46)	2.71 (1.05–4.38)	6.58 (198.24)	<0.001
Selinexor	Oncological medication	11.44 (8.79–14.88)	11.36 (11.1–11.62)	11.32 (9.08–14.11)	3.5 (1.83–5.17)	11.36 (527.18)	<0.001
Tamoxifen	Oncological medication	13.39 (9.29–19.31)	13.28 (12.91–13.64)	13.25 (9.76–18)	3.73 (2.06–5.4)	13.28 (328.78)	<0.001
Aflibercept	Ophthalmic medication	12.86 (11.34–14.57)	12.76 (12.63–12.88)	12.55 (11.3–13.93)	3.65 (1.98–5.32)	12.76 (2672.98)	<0.001
Brolucizumab	Ophthalmic medication	8.04 (5.84–11.06)	8 (7.68–8.32)	7.98 (6.11–10.42)	3 (1.33–4.66)	8 (232.24)	<0.001
Faricimab	Ophthalmic medication	7.29 (4.96–10.73)	7.26 (6.88–7.65)	7.25 (5.25–10.01)	2.86 (1.19–4.52)	7.26 (140.28)	<0.001
Omidenepag isopropyl	Ophthalmic medication	238.95 (92.9–614.61)	205.9 (205.09–206.72)	205.83 (93.37–453.75)	7.69 (5.95–9.42)	205.9 (1019.87)	<0.001
Travoprost	Ophthalmic medication	9.3 (5.39–16.04)	9.24 (8.7–9.79)	9.24 (5.85–14.58)	3.21 (1.54–4.87)	9.24 (95.55)	<0.001
Verteporfin	Ophthalmic medication	6.27 (3.55–11.05)	6.25 (5.68–6.81)	6.24 (3.88–10.03)	2.64 (0.98–4.31)	6.25 (52.87)	<0.001
Icatibant	Other medication	4.73 (1.77–12.63)	4.72 (3.74–5.7)	4.72 (2.08–10.73)	2.24 (0.57–3.91)	4.72 (11.73)	0.004
Salmeterol	Other medication	6.52 (3.39–12.56)	6.5 (5.85–7.15)	6.5 (3.76–11.24)	2.7 (1.03–4.37)	6.5 (41.89)	<0.001
Abatacept	Other medication	3.98 (3.39–4.66)	3.97 (3.81–4.13)	3.94 (3.45–4.5)	1.98 (0.31–3.64)	3.97 (340.81)	<0.001
Alprostadil	Other medication	6.83 (2.2–21.22)	6.8 (5.67–7.93)	6.8 (2.63–17.56)	2.77 (1.1–4.43)	6.8 (14.85)	0.002
Belumosudil	Other medication	11.72 (5.57–24.66)	11.64 (10.9–12.37)	11.63 (6.24–21.67)	3.54 (1.87–5.21)	11.64 (68.06)	<0.001
Cinacalcet	Other medication	6.78 (3.04–15.11)	6.75 (5.95–7.55)	6.75 (3.45–13.2)	2.75 (1.09–4.42)	6.75 (29.39)	<0.001
D-alpha-tocopherol acetate	Other medication	4.34 (2.07–9.13)	4.33 (3.6–5.07)	4.33 (2.33–8.06)	2.12 (0.45–3.78)	4.33 (17.96)	<0.001
Deflazacort	Other medication	7.02 (4.57–10.78)	6.99 (6.56–7.42)	6.98 (4.87–9.99)	2.8 (1.14–4.47)	6.99 (107.67)	<0.001
Eltrombopag	Other medication	3.62 (2.59–5.04)	3.61 (3.28–3.94)	3.6 (2.73–4.76)	1.85 (0.18–3.52)	3.61 (65.89)	<0.001
Etelcalcetide	Other medication	8.21 (4.85–13.88)	8.17 (7.65–8.69)	8.16 (5.26–12.67)	3.03 (1.36–4.7)	8.17 (88.05)	<0.001
Fenofibrate	Other medication	8.29 (2.67–25.79)	8.25 (7.12–9.38)	8.25 (3.19–21.32)	3.04 (1.37–4.71)	8.25 (19.12)	<0.001
Hydrochlorothiazide	Other medication	5.65 (3.75–8.51)	5.63 (5.22–6.04)	5.62 (3.99–7.92)	2.49 (0.83–4.16)	5.63 (87.52)	<0.001
Hydroxychloroquine	Other medication	3.84 (2.95–5.01)	3.84 (3.57–4.1)	3.83 (3.06–4.78)	1.94 (0.27–3.6)	3.84 (115)	<0.001
Ivacaftor	Other medication	4.27 (2.13–8.55)	4.26 (3.57–4.95)	4.26 (2.38–7.61)	2.09 (0.42–3.76)	4.26 (19.97)	<0.001
Lumacaftor	Other medication	3.21 (1.96–5.24)	3.2 (2.71–3.69)	3.2 (2.12–4.82)	1.68 (0.01–3.34)	3.2 (24.22)	<0.001
Nitisinone	Other medication	68.22 (50.57–92.02)	65.27 (64.98–65.55)	65.06 (50.65–83.58)	6.02 (4.36–7.69)	65.27 (2840.56)	<0.001
Omega-3-acid ethyl esters	Other medication	30.54 (20.71–45.05)	29.95 (29.57–30.33)	29.89 (21.59–41.38)	4.9 (3.23–6.57)	29.95 (726.61)	<0.001
Sevelamer	Other medication	20.5 (7.64–55.01)	20.24 (19.26–21.21)	20.23 (8.86–46.2)	4.34 (2.66–6.01)	20.24 (73.18)	<0.001
Sildenafil	Other medication	3.9 (2.63–5.77)	3.89 (3.5–4.28)	3.88 (2.79–5.39)	1.96 (0.29–3.62)	3.89 (53.56)	<0.001
Tamsulosin	Other medication	16.03 (13.17–19.52)	15.87 (15.68–16.07)	15.76 (13.37–18.59)	3.98 (2.31–5.64)	15.87 (1398.16)	<0.001
Tezacaftor	Other medication	4 (2.98–5.36)	3.99 (3.7–4.28)	3.98 (3.12–5.09)	1.99 (0.33–3.66)	3.99 (100.63)	<0.001
Tolterodine	Other medication	5.49 (2.74–11)	5.48 (4.78–6.17)	5.47 (3.06–9.79)	2.45 (0.79–4.12)	5.48 (29.27)	<0.001
Upadacitinib	Other medication	3.86 (2.84–5.25)	3.85 (3.55–4.16)	3.85 (2.97–4.97)	1.94 (0.28–3.61)	3.85 (86.47)	<0.001

The *p*-value represents the statistical test value from the Chi-square test in the PRR algorithm. CI, confidence interval; ROR, reporting odds ratio; PRR, proportional reported ratio; BCPNN, Bayesian confidence propagation neural network; MGPS, multi-item gamma Poisson shrinker.

### Risk values of drugs associated with drug-induced cataract

**Figure 3:**
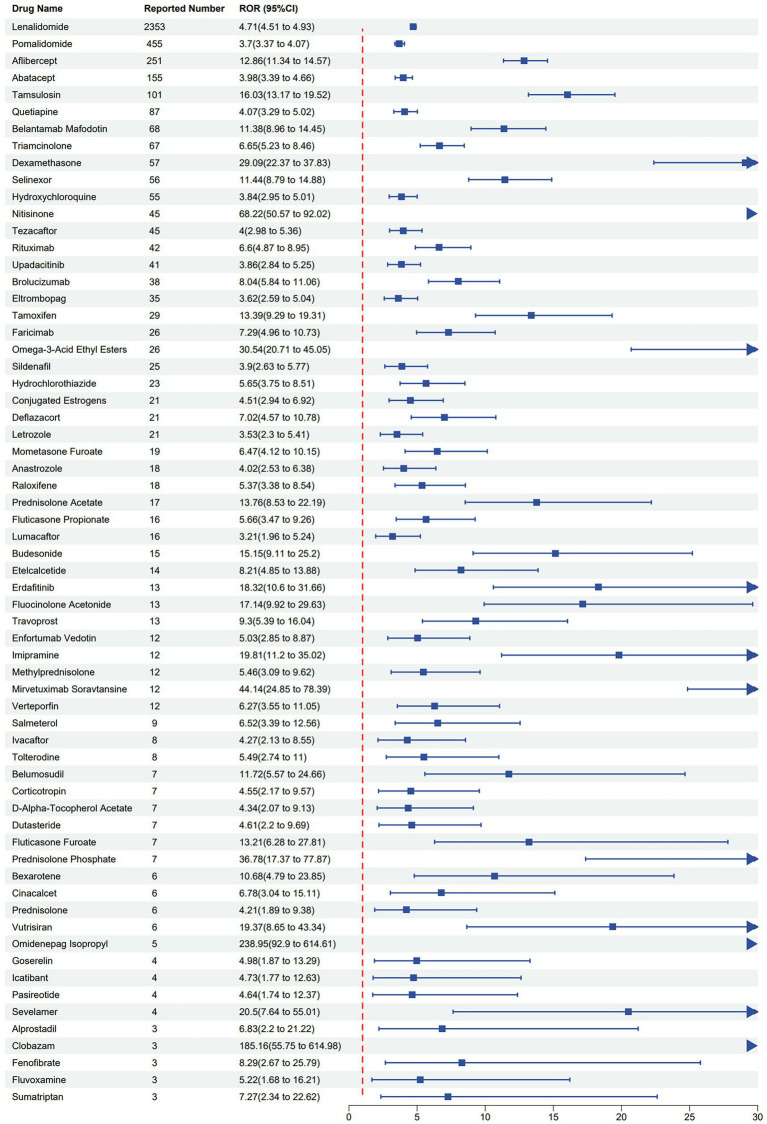
Forest plot of ROR-based positive signals for drug-induced cataract from the FAERS database.

Among the drugs associated with cataract, the top three hormonal medications by ROR values were prednisolone phosphate (ROR = 36.78), dexamethasone (ROR = 29.09), and budesonide (ROR = 15.15). In the oncological class, mirvetuximab soravtansine showed the strongest cataract signal (ROR = 44.14), followed by erdafitinib (ROR = 18.32) and tamoxifen (ROR = 13.39). For ophthalmic medications, the top three drugs by ROR values were omidenepag isopropyl (ROR = 238.95), aflibercept (ROR = 12.86), and brolucizumab (ROR = 8.04). In other medication classes, the top three drugs by ROR values were nitisinone (ROR = 68.22), Omega-3-Acid Ethyl Esters (ROR = 30.54), and tamsulosin (ROR = 16.03). Further details are provided in Table 4 and Figure 3.

### Risk, reporting frequency of drugs

**Figure 4:**
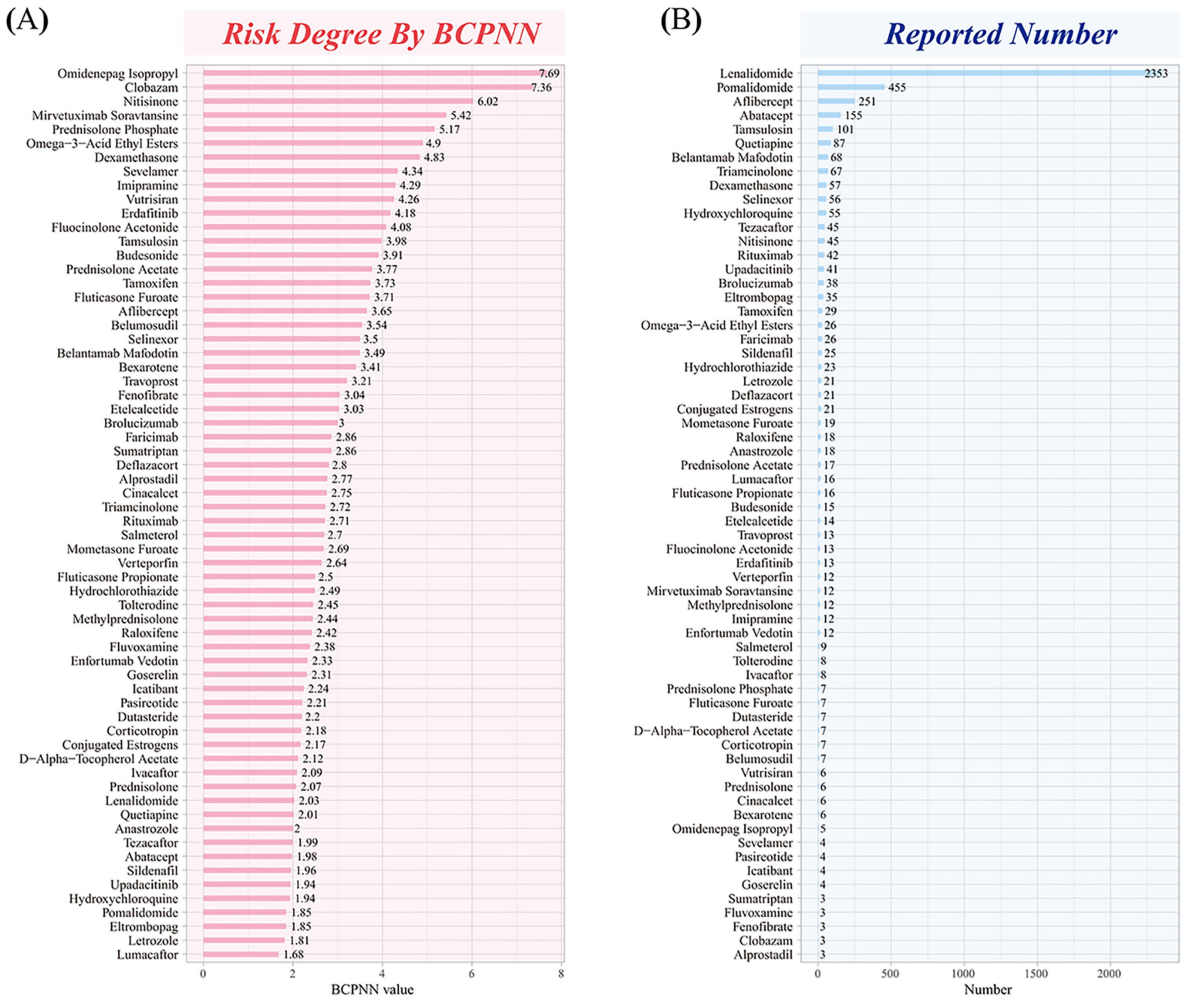
Distribution of risk and case count for drug-induced cataract. **(**A**)** Signals ranking of drugs associated with cataract. **(**B**)** Frequency of adverse event reports.

The BCPNN algorithm was utilized to assess the risk of drug-induced cataract, with 26 drugs (40.6%) classified as high-risk and 38 drugs (59.4%) classified as medium-risk. The top three drugs with the highest risk levels were omidenepag isopropyl (IC_025_ = 7.69), clobazam (IC_025_ = 7.36), and nitisinone (IC_025_ = 6.02). Conversely, the three drugs with the lowest risk levels were lumacaftor (IC_025_ = 1.68), letrozole (IC_025_ = 1.81), and eltrombopag (IC_025_ = 1.85). Based on the frequency of adverse event reports, Figure 4 lists drugs associated with cataract, with lenalidomide (*n* = 2,353) having the highest number of reports, followed by pomalidomide (*n* = 455), aflibercept (*n* = 251), abatacept (*n* = 155), and tamsulosin (*n* = 101).

### Comparison of drug-induced onset time among different categories of drugs

**Figure 5:**
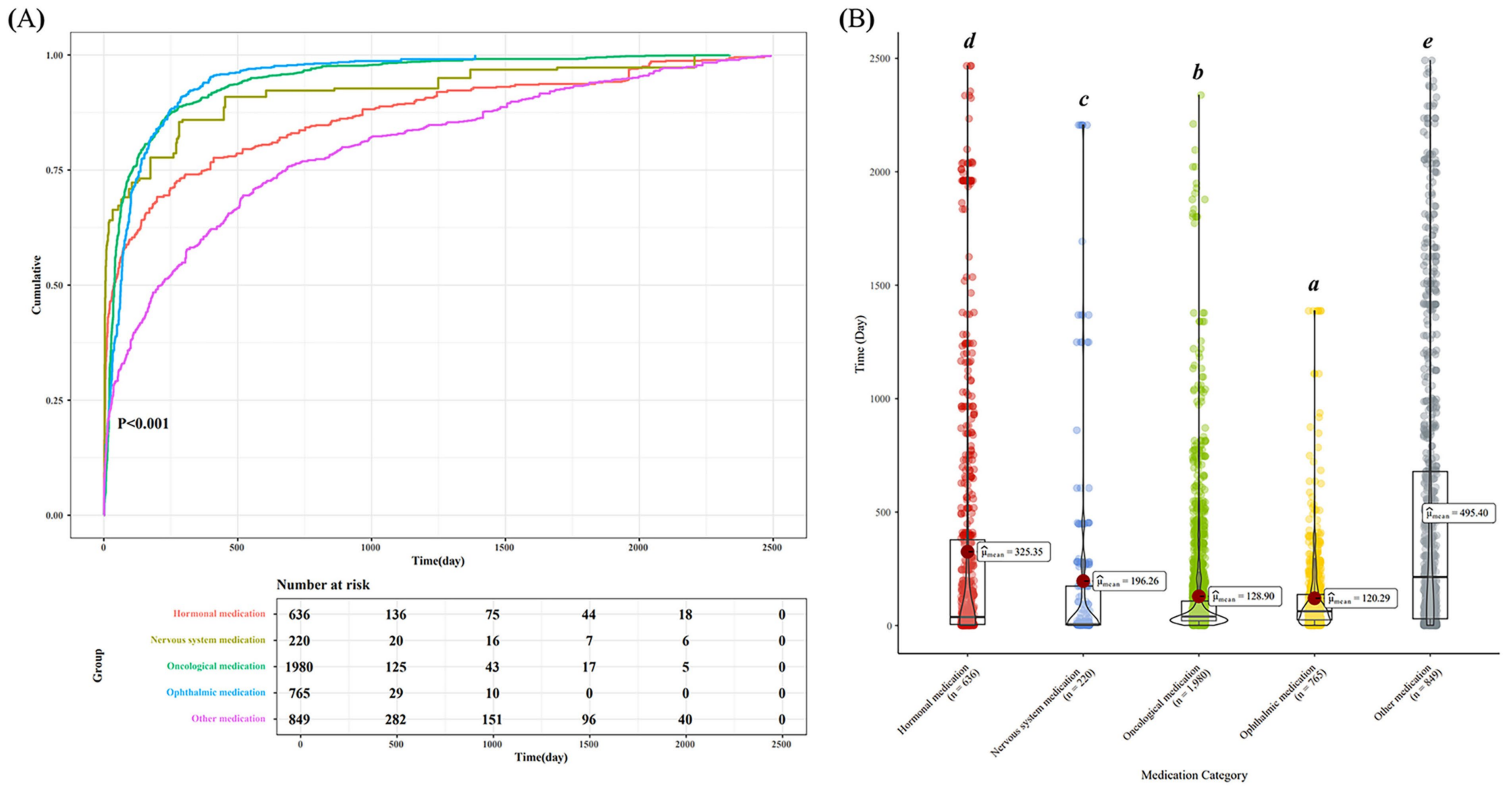
Onset time of adverse reactions in drug-induced cataract. **(A)** Analysis of cumulative reports of drug-related cataract across drug classes over time. **(B)** Median onset times differed across drug classifications.

One-way ANOVA revealed significant differences in the time-to-onset of drug-induced cataract across therapeutic classes (*p* < 0.001). Ophthalmic medications had the shortest onset time (mean = 120.29 days), followed by oncological drugs (mean = 128.9 days), nervous system medications (mean = 196.26 days), and hormonal therapies (mean = 325.35 days). Other medications exhibited the longest progression time (mean = 495.40 days) (Figure 5).

### Multivariate analysis results

**Figure 6:**
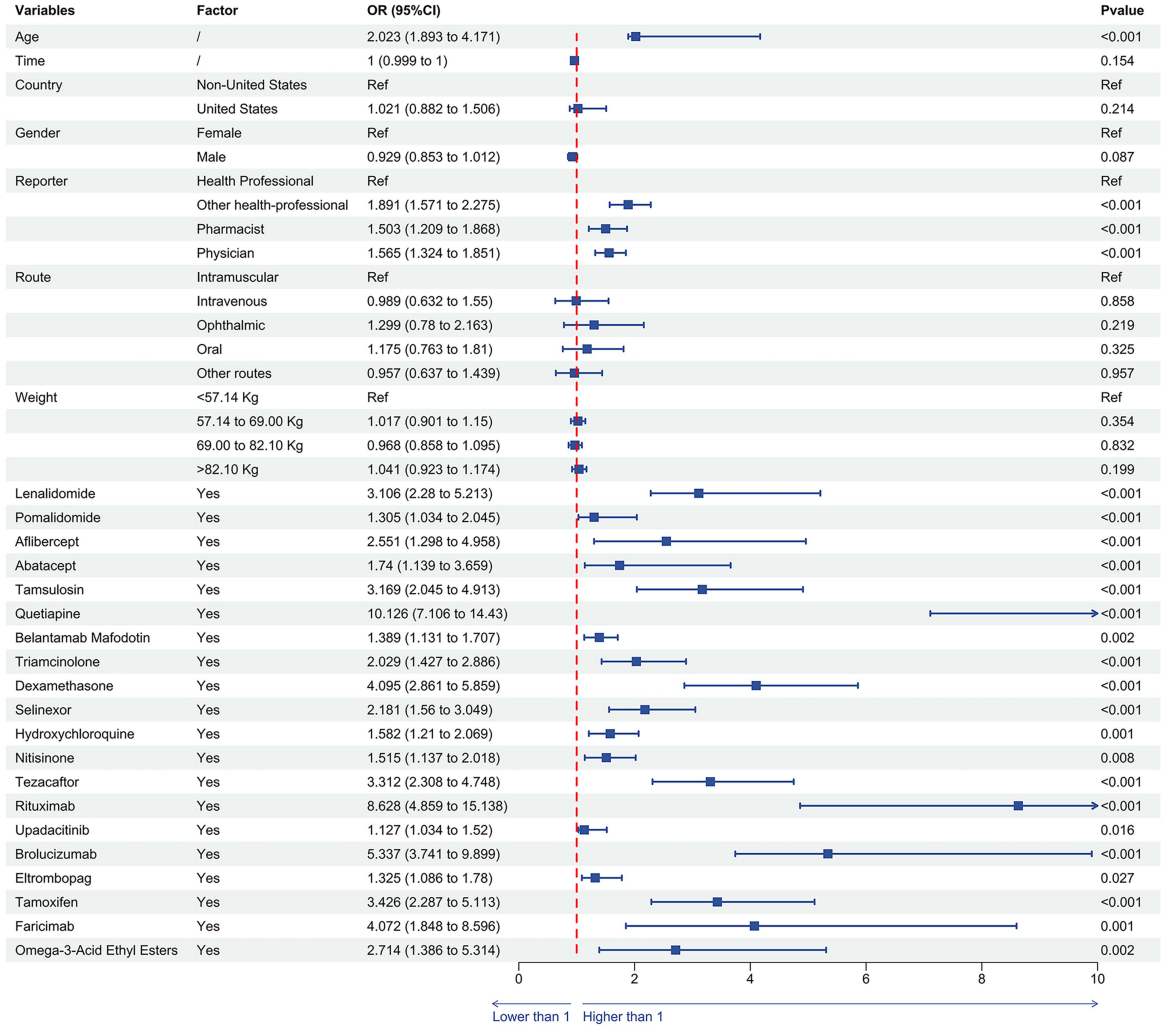
Multivariate logistic regression results of the top 20 drugs associated with cataract adverse event reports. OR, odds ratio.

Furthermore, we incorporated comprehensive data for all subjects exposed to the top 20 signal drugs—including age, weight, gender, country, drug indications, reporter type, route of administration, and duration of use—into a multivariable logistic regression model. The analysis confirmed that each of the top 20 drugs remained an independent risk factor for drug-associated cataract (OR >1, *p* < 0.05). Additionally, older age was identified as a significant risk factor for cataract development [OR (95% CI) = 2.023 (1.893–4.171)]. Regarding reporting sources, compared with reports submitted by “Health Professional,” those from “Other Health-Professional” [OR (95% CI) = 1.891 (1.571–2.275)], “Pharmacist” [OR (95% CI) = 1.503 (1.209–1.868)], and “Physician” [OR (95% CI) = 1.565 (1.324–1.851)] were associated with a significantly higher likelihood of cataract reporting (*p* < 0.05) (Figure 6).

## Discussion

This study systematically evaluated drug-induced cataract reports in the FAERS database from January 2004 to December 2024. Disproportionality analysis identified 64 medications with significant positive signals, predominantly across five therapeutic classes: hormonal agents, oncological drugs, ophthalmic medications, nervous system medications, and other pharmacological categories. The associated risks and onset times for these drugs were also assessed. Additionally, subgroup analyses were conducted stratified by age, sex, reporting country, and underlying medical conditions. These findings provide critical insights for reducing cataract prevalence and improving drug safety, while assisting ophthalmologists in identifying cataract-inducing medications and mitigating the risk of drug-induced cataract.

Our analysis shows a clear increase in reports of drug-induced cataract from 2004 to 2019, followed by a decrease after that (Figure 2B). This trend can be explained by several related factors. The initial rise likely resulted from greater awareness among clinicians and increased use of medications in growing older populations. Elderly patients often take multiple medications long-term and are more prone to cataract, which may have further contributed to the upward trend (2).

The decline after 2019, however, requires careful interpretation. It could reflect better drug safety or improved prescribing habits, but it also coincides with the COVID-19 pandemic. During this period, reduced non-urgent medical visits—including eye examinations—likely led to fewer cases being detected and reported (13). Interestingly, reports of drug-induced cataract were more common in female patients. This difference may be due to several reasons. Certain high-risk drugs, such as hormonal treatments (e.g., tamoxifen) and medications for autoimmune conditions like rheumatoid arthritis, are prescribed more frequently to women. Additionally, women may be more likely to seek medical care, leading to higher detection and reporting rates.

Our analysis found that ophthalmic medications had the shortest mean onset time for drug-induced cataract (120.29 days). This earlier onset may be attributed to two main factors. First, the local route of administration bypasses the blood-retinal barrier, allowing direct and sustained high drug concentrations at the ocular site (14). Second, patients on these therapies typically undergo more frequent ocular evaluations, which facilitates earlier detection of cataract formation. Anti-VEGF agents and prostaglandin analogs emerged with cataract risk signals in our study. While intravitreal injections carry a known procedural risk of mechanical lens injury (15), drug-specific effects are also likely involved. For anti-VEGF agents, cataract formation may result from complex biochemical alterations within the vitreous cavity that disrupt the lens microenvironment. One critical pathway implicated in this process is the c-Src/VEGF pathway, which is known to be activated under oxidative stress conditions (16). Zhang et al. demonstrated that oxidative stress can lead to increased VEGF expression and activation of c-Src kinase in lens epithelial cells (17). Among prostaglandin analogs, omidenepag isopropyl demonstrated the strongest pharmacovigilance signal (ROR = 238.95, IC_025_ = 7.69). This finding aligns with earlier FAERS-based research linking another prostaglandin analog, latanoprost, to drug-induced cataract, suggesting a potential class effect (17). Both omidenepag isopropyl and travoprost are effective ocular hypotensive agents used in glaucoma management, with known adverse effects including conjunctival hyperemia, corneal thickening, macular edema, and ocular inflammation (18, 19). However, drug-induced cataract have not been directly reported in clinical studies for these medications. An important consideration is that the higher frequency of eye examinations in patients using ophthalmic medications may increase cataract detection rates, introducing a potential surveillance bias. Therefore, further research is necessary to clarify the precise mechanisms and better characterize the cataract risk associated with these therapeutic agents.

Corticosteroids are widely utilized in clinical practice, both as topical agents in ophthalmology and as systemic treatments for conditions such as asthma, arthritis, leukemia, and nephrotic syndrome (20, 21). Despite their potent anti-inflammatory and immunosuppressive effects, corticosteroids are well-documented to carry a significant risk of cataract formation, particularly with high-dose or long-term use. Our study further supports this association, identifying significant safety signals for drugs such as dexamethasone, prednisolone phosphate, and budesonide. Glucocorticoid-induced cataract typically present as central, posterior subcapsular opacities accompanied by vacuoles, a pathological hallmark indicative of abnormal migration and differentiation of lens epithelial cells (LECs) along the posterior capsule (22). With an estimated 1% of the US population undergoing long-term glucocorticoid therapy (23), this adverse effect represents a considerable public health concern, emphasizing the need for vigilant monitoring and risk mitigation in clinical practice.

Our findings indicate that, following hormonal agents, oncological agents are associated with the highest incidence of drug-induced cataract. Mirvetuximab soravtansine, a folate receptor alpha-directed antibody and microtubule inhibitor conjugate used to treat various types of treatment-resistant cancers (24), carries a risk of cataract formation, with an ROR of 44.14. Ocular toxicities are common treatment-related adverse events with mirvetuximab soravtansine (25). A study reported that 20% of patients required dose reductions due to adverse events, with the most frequent causes being visual impairment (9%), keratopathy (7%), and cataract (3%) (26). Although the exact mechanism remains unclear, the potential cataract risk associated with mirvetuximab soravtansine necessitates clinical vigilance. Erdafitinib, a fibroblast growth factor receptor (FGFR) inhibitor used to treat locally advanced or metastatic urothelial carcinoma, has also been associated with cataract risk. A pharmacovigilance study utilizing the FAERS database identified a significant association between erdafitinib treatment and visual impairment, with an ROR of 3.49 (95% CI, 1.93–6.30) (27). The FGFR signaling pathway is directly involved in the proliferation of LECs (28, 29), suggesting that FGFR inhibition may disrupt ionic balance and cellular renewal in the lens, potentially leading to cataract formation. Tamoxifen, a selective estrogen receptor modulator commonly prescribed for breast cancer therapy (30), has been linked to an increased risk of cataract. Gorin et al. (31) reported a 4.03-fold higher risk of posterior subcapsular cataract formation associated with tamoxifen use. The proposed mechanism involves tamoxifen-induced dysfunction of chloride channels in LECs, disrupting electrolyte balance and leading to lens opacification (32). Additionally, other anticancer agents, such as belantamab mafodotin, selinexor, and bexarotene, also pose a significant pharmacological risk for cataract formation. Given that cancer patients typically require extended and regular medication as part of their treatment regimen, ophthalmic evaluation—including visual acuity tests, slit-lamp examination, and fundoscopy—should be conducted before initiating therapy with these agents. Regular follow-up during treatment is essential, and immediate referral to ophthalmology is necessary if visual disturbances.

Among nervous system medications, six drugs showed positive signals for inducing cataract. Our study confirms that imipramine, fluvoxamine, and quetiapine, psychotropic drugs, are associated with cataract formation, consistent with existing clinical reports and epidemiological studies on conventional antipsychotics (33). While direct causal pathways remain insufficiently understood, one proposed mechanism is that antipsychotics disrupt signaling systems, promoting protein aggregation within the lens and predisposing individuals to cataract formation (34, 35). Clobazam, a benzodiazepine derivative, is used in managing severe forms of epilepsy (36). Vutrisiran, a novel therapy, halts hereditary transthyretin-mediated amyloidosis to prevent progressive neuropathy (37). Sumatriptan, a serotonin receptor agonist, is used for acute intervention in migraine and cluster headache attacks (38). The current available clinical data do not allow for a comprehensive assessment of the causal relationship between these drugs and cataract formation, highlighting the need for more in-depth studies on the association between nervous system medications and cataract.

Other drugs, including nitisinone, Omega-3-Acid Ethyl Esters, and tamsulosin, have also been implicated in cataract formation, further expanding the list of medications associated with cataract. Nitisinone, used to treat hereditary tyrosinemia type 1 (HT-1) and alkaptonuria, has been reported to cause cataract as an adverse effect (39). One potential mechanism involves nitisinone therapy, which raises tyrosine levels, depletes glutathione, impairs antioxidant defenses, and results in increased oxidative stress, ultimately leading to cataract formation (40). Antilipemic agents like Omega-3-Acid Ethyl Esters and fenofibrate, used to lower cholesterol and fat levels in the blood, are also linked to a high pharmacological risk for drug-induced cataract. Elderly patients taking these medications should undergo regular lens examinations to monitor for any changes (41). Additionally, andrological medications such as tamsulosin and alprostadil were identified in this study as having a significant risk for drug-induced cataract. Our findings contribute valuable insights into the risk of drug-induced cataract and provide a foundation for future research on the underlying mechanisms.

Given these findings, a wide range of medications are associated with cataract formation, and clinicians should remain vigilant for the possibility of drug-induced cataract. For patients requiring these medications due to underlying health conditions, physicians should proactively discuss the potential risks, closely monitor treatment responses, and consider arranging periodic ophthalmologic evaluations after starting therapy.

### Limitations

This study has several limitations. Firstly, while disproportionality analysis can detect potential drug–event associations, it does not establish causality. Secondly, unmeasured confounding factors—such as age, comorbidities, or concomitant medication use—may influence the observed relationships. Thirdly, although subgroup analyses were performed, the FAERS database relies on voluntary and spontaneous reporting, which is subject to underreporting, reporting delays, inaccuracies, and incomplete data; these inherent limitations may bias disproportionality estimates. Finally, external validation in broader populations is needed to confirm the robustness of our findings.

## Conclusion

In conclusion, our findings comprehensively confirm the demographic and epidemiological characteristics of drug-induced cataract and identify specific medications with significant safety signals. By systematically classifying these drugs based on therapeutic class, risk magnitude, and time-to-onset, our study offers critical insights for clinical practice and provides an essential safety assessment of these medications.

## Data Availability

The raw data supporting the conclusions of this article will be made available by the authors, without undue reservation.

## References

[1] Gallo Afflitto G Aiello F Surico PL Malek DA Mori T Swaminathan SS . Cataract and risk of fracture: a systematic review, meta-analysis, and Bayesian network meta-analysis. Ophthalmology. (2025) 132:921–34. doi: 10.1016/j.ophtha.2025.02.010, 39978438

[2] Chen SP Woreta F Chang DF. Cataracts: a review. JAMA. (2025) 333:2093–103. doi: 10.1001/jama.2025.1597, 40227658

[3] Vision Loss Expert Group of the Global Burden of Disease Study, GBD 2019 Blindness and Vision Impairment Collaborators. Global estimates on the number of people blind or visually impaired by cataract: a meta-analysis from 2000 to 2020. Eye (Lond). (2024) 38:2156–72. doi: 10.1038/s41433-024-02961-1 38461217 PMC11269584

[4] Li C Lu Y Chen M Zhang Q Zhang Z Xi W . Dietary-related characteristics and cataract risk: evidence from a mendelian randomization study. Exp Biol Med (Maywood). (2025) 250:10544. doi: 10.3389/ebm.2025.10544 40831670 PMC12358322

[5] Gong D Ma DH Zhang Q Dang KR Yang WH Wang JT. Risk prediction model for cataract after vitrectomy surgery: a 2-year study on primary rhegmatogenous retinal detachment. Int J Ophthalmol. (2025) 18:2106–15. doi: 10.18240/ijo.2025.11.12, 41158177 PMC12554527

[6] Li X Wang SW Zhang ZJ Luo ZY Tang JF Tao T. Real-world pharmacovigilance analysis of drug-related cataracts using the FDA adverse event reporting system database. Front Pharmacol. (2025) 16:1498191. doi: 10.3389/fphar.2025.1498191 40343006 PMC12058479

[7] Carlson J McBride K O’Connor M. Drugs associated with cataract formation represent an unmet need in cataract research. Front Med. (2022) 9:947659. doi: 10.3389/fmed.2022.947659 PMC9420850 36045926

[8] Potter E Reyes M Naples J Pan GD. FDA adverse event reporting system (FAERS) essentials: a guide to understanding, applying, and interpreting adverse event data reported to FAERS. Clin Pharmacol Ther. (2025) 118:567–82. doi: 10.1002/cpt.3701, 40384638 PMC12393772

[9] Xiao K Chen X Wu S Zhang Y Chen R Wu H . Real-world large sample evaluation of drug-related blepharoptosis: a pharmacovigilance analysis of the FDA adverse event reporting system database. Ther Adv Drug Saf. (2025) 16:1983. doi: 10.1177/20420986251371983 PMC12454954 40995193

[10] Yokotsuka M Aoyama M Kubota K. The use of a medical dictionary for regulatory activities terminology (MedDRA) in prescription-event monitoring in Japan (J-PEM). Int J Med Inform. (2000) 57:139–53. doi: 10.1016/S1386-5056(00)00062-9, 10961570

[11] Kinoshita S Hosomi K Yokoyama S Takada M. Time-to-onset analysis of amiodarone-associated thyroid dysfunction. J Clin Pharm Ther. (2020) 45:65–71. doi: 10.1111/jcpt.13024, 31400296

[12] He CZ Qiu Q Lu SJ Xue FL Liu JQ He Y. Adverse event reporting of faricimab: a disproportionality analysis of FDA adverse event reporting system (FAERS) database. Front Pharmacol. (2025) 16:1521358. doi: 10.3389/fphar.2025.1521358 40144657 PMC11936923

[13] Jeong E Nelson SD Su Y Malin B Li L Chen Y. Detecting drug-drug interactions between therapies for COVID-19 and concomitant medications through the FDA adverse event reporting system. Front Pharmacol. (2022) 13:938552. doi: 10.3389/fphar.2022.938552 35935872 PMC9353301

[14] Samoilă L Voștinaru O Dinte E Bodoki AE Iacob BC Bodoki E . Topical treatment for retinal degenerative pathologies: a systematic review. Int J Mol Sci. (2023) 24:8045. doi: 10.3390/ijms24098045 37175752 PMC10178888

[15] Uludag G Hassan M Matsumiya W Pham BH Chea S Trong Tuong Than N . Efficacy and safety of intravitreal anti-VEGF therapy in diabetic retinopathy: what we have learned and what should we learn further? Expert Opin Biol Ther. (2022) 22:1275–91. doi: 10.1080/14712598.2022.2100694, 35818801 PMC10863998

[16] Fu L Yang Q Han Y Sun F Jin J Wang J. Slit2 promotes H2O2-induced lens epithelial cells oxidative damage and age-related cataract. Curr Eye Res. (2025) 50:41–50. doi: 10.1080/02713683.2024.2388698, 39143744

[17] Zhang L Zhang ZF Hui YN He F Guan XR Zhou J. Oxidative stress participates in age-related cataract formation by disrupting connection between lens epithelial cells through c-src/VEGF pathway. Curr Eye Res. (2024) 49:380–90. doi: 10.1080/02713683.2023.2293456, 38108278

[18] Sarkisian SR Ang RE Lee AM Berdahl JP Heersink SB Burden JH . Phase 3 randomized clinical trial of the safety and efficacy of Travoprost intraocular implant in patients with open-angle glaucoma or ocular hypertension. Ophthalmology. (2024) 131:1021–32. doi: 10.1016/j.ophtha.2024.02.022, 38423216

[19] Matsuo M Matsuoka Y Tanito M. Efficacy and patient tolerability of Omidenepag isopropyl in the treatment of glaucoma and ocular hypertension. Clin. Ophthalmol. (2022) 16:1261–79. doi: 10.2147/OPTH.S340386, 35510270 PMC9058248

[20] Liu H Ji M Xiao P Gou J Yin T He H . Glucocorticoids-based prodrug design: current strategies and research progress. Asian J Pharm Sci. (2024) 19:100922. doi: 10.1016/j.ajps.2024.100922, 38966286 PMC11222810

[21] Christian MT Maxted AP. Optimizing the corticosteroid dose in steroid-sensitive nephrotic syndrome. Pediatr Nephrol. (2022) 37:37–47. doi: 10.1007/s00467-021-04985-1, 33611671 PMC7896825

[22] Zhang Y Si W Mao Y Xu S Li F Liu J . Upregulation of ferroptosis in glucocorticoids-induced posterior subcapsular cataracts. Commun Biol. (2025) 8:613. doi: 10.1038/s42003-025-08067-y, 40234585 PMC12000516

[23] Humphrey MB Russell L Danila MI Fink HA Guyatt G Cannon M . 2022 American College of Rheumatology Guideline for the prevention and treatment of Glucocorticoid-Induced osteoporosis. Arthritis & Rheumatology. (2023) 75:2088–102. doi: 10.1002/art.42646, 37845798

[24] Moore KN Angelergues A Konecny GE García Y Banerjee S Lorusso D . Mirvetuximab Soravtansine in FRα-positive, platinum-resistant ovarian cancer. N Engl J Med. (2023) 389:2162–74. doi: 10.1056/NEJMoa2309169, 38055253

[25] Zhu Y Liu K Wang K Zhu H. Treatment-related adverse events of antibody–drug conjugates in clinical trials: a systematic review and meta-analysis. Cancer. (2023) 129:283–95. doi: 10.1002/cncr.34507, 36408673 PMC10099922

[26] Dilawari A Shah M Ison G Gittleman H Fiero MH Shah A . FDA approval summary: Mirvetuximab soravtansine-gynx for FRα-positive, platinum-resistant ovarian cancer. Clin Cancer Res. (2023) 29:3835–40. doi: 10.1158/1078-0432.CCR-23-0991, 37212825 PMC10592645

[27] Yuan T Li F Hou Y Guo H. Adverse events in patients with advanced urothelial carcinoma treated with erdafitinib: a retrospective pharmacovigilance study. Front Pharmacol. (2023) 14:1266890. doi: 10.3389/fphar.2023.1266890 38074150 PMC10702547

[28] Zhao H Yang T Madakashira BP Thiels CA Bechtle CA Garcia CM . Fibroblast growth factor receptor signaling is essential for lens fiber cell differentiation. Dev Biol. (2008) 318:276–88. doi: 10.1016/j.ydbio.2008.03.028, 18455718 PMC2574794

[29] Padula SL Sidler EP Wagner BD Manz CJ Lovicu FJ Robinson ML. Lens fiber cell differentiation occurs independently of fibroblast growth factor receptor signaling in the absence of Pten. Dev Biol. (2020) 467:1–13. doi: 10.1016/j.ydbio.2020.07.017, 32858001 PMC7572784

[30] Kim H Whitman AA Wisniewska K Kakati RT Garcia-Recio S Calhoun BC . Tamoxifen response at single-cell resolution in estrogen receptor-positive primary human breast tumors. Clin Cancer Res. (2023) 29:4894–907. doi: 10.1158/1078-0432.CCR-23-1248, 37747807 PMC10690085

[31] Gorin MB Day R Costantino JP Fisher B Redmond CK Wickerham L . Long-term tamoxifen citrate use and potential ocular toxicity. Am J Ophthalmol. (1998) 125:493–501. doi: 10.1016/S0002-9394(99)80190-1, 9559735

[32] Zhang JJ Jacob TJ Valverde MA Hardy SP Mintenig GM Sepúlveda FV . Tamoxifen blocks chloride channels. A possible mechanism for cataract formation. J Clin Invest. (1994) 94:1690–7. doi: 10.1172/JCI117514, 7929848 PMC295332

[33] Chu CS Chou PH Chen YH Huang MW Hsu MY Lan TH . Association between antipsychotic drug use and cataracts in patients with bipolar disorder: a population-based, nested case-control study. J Affect Disord. (2017) 209:86–92. doi: 10.1016/j.jad.2016.11.019, 27889598

[34] Shahzad S Suleman MI Shahab H Mazour I Kaur A Rudzinskiy P . Cataract occurrence with antipsychotic drugs. Psychosomatics. (2002) 43:354–9. doi: 10.1176/appi.psy.43.5.354, 12297603

[35] Moreau KL King JA. Protein misfolding and aggregation in cataract disease and prospects for prevention. Trends Mol Med. (2012) 18:273–82. doi: 10.1016/j.molmed.2012.03.005, 22520268 PMC3621977

[36] Stephen LJ Brodie MJ. Pharmacological management of the genetic generalised epilepsies in adolescents and adults. CNS Drugs. (2020) 34:147–61. doi: 10.1007/s40263-020-00698-5, 31983023

[37] Karimi MA Esmaeilpour Moallem F Gholami Chahkand MS Azarm E Emami Kazemabad MJ Dadkhah PA. Assessing the effectiveness and safety of patisiran and vutrisiran in ATTRv amyloidosis with polyneuropathy: a systematic review. Front Neurol. (2024) 15:1465747. doi: 10.3389/fneur.2024.1465747 39286810 PMC11402727

[38] Wu JW Lai PY Chen YL Wang YF Lirng JF Chen ST . The use of neuroimaging for predicting sumatriptan treatment response in patients with migraine. Front Neurol. (2022) 13:798695. doi: 10.3389/fneur.2022.798695 35173673 PMC8841861

[39] Lock EA. The discovery of the mode of action of nitisinone. Meta. (2022) 12:902 10.3390/metabo12100902 PMC9609752 36295804

[40] Ahmad MSZ Ahmed M Khedr M Borgia A Madden A Ranganath LR . Association of alkaptonuria and low dose nitisinone therapy with cataract formation in a large cohort of patients. JIMD Rep. (2022) 63:351–60. doi: 10.1002/jmd2.12288, 35822094 PMC9259401

[41] Khan H Aftab OM Billah MS Khouri AS. Beyond the prostate: a visionary study on ocular impacts of benign prostatic hyperplasia drugs. J Ocul Pharmacol Ther. (2025) 41:475–84. doi: 10.1089/jop.2025.0030 40764034

